# Pathobiology of tobacco smoking and neurovascular disorders: untied strings and alternative products

**DOI:** 10.1186/s12987-015-0022-x

**Published:** 2015-10-31

**Authors:** Pooja Naik, Luca Cucullo

**Affiliations:** Department of Pharmaceutical Sciences, Texas Tech University Health Sciences Center, School of Pharmacy, 1300 S. Coulter Street, Amarillo, TX 79106 USA; Center for Blood Brain Barrier Research, Texas Tech University Health Sciences Center, Amarillo, TX 79106 USA

**Keywords:** Blood–brain barrier, Cerebrovascular disease, Nrf2, Antioxidant response, Electronic cigarettes, Inflammation, Alternatives, Free radical, Stroke, Diabetes

## Abstract

Tobacco smoke (TS) is the leading cause of preventable deaths worldwide. In addition to a host of well characterized diseases including chronic obstructive pulmonary disease, oral and peripheral cancers and cardiovascular complications, epidemiological evidence suggests that chronic smokers are at equal risk to develop neurological and neurovascular complications such as multiple sclerosis, Alzheimer’s disease, stroke, vascular dementia and small vessel ischemic disease (SVID). Unfortunately, few direct neurotoxicology studies of tobacco smoking and its pathogenic pathways have been produced so far. A major link between TS and CNS disorders is the blood–brain barrier (BBB). In this review article, we summarize the current understanding of the toxicological impact of TS on BBB physiology and function and major compensatory mechanisms such as nrf2- ARE signaling and anti-inflammatory pathways activated by TS. In the same context, we discuss the controversial role of antioxidant supplementation as a prophylactic and/or therapeutic approach in delaying or decreasing the disease complications in smokers. Further, we cover a number of toxicological studies associated with “reduced exposure” cigarette products including electronic cigarettes. Finally, we provide insights on possible avenues for future research including mechanistic studies using direct inhalation rodent models.

## Background

Tobacco smoke (TS) is the leading cause of preventable death, accounting for more than 6 million premature annual deaths worldwide and over 480,000/year in the United States alone [1, 2]. Smoking and other tobacco use almost always begins at a young age and a large percentage continue to smoke as adults, becoming lifelong smokers. Currently about 5.6 million youths between 0 and 17 years of age are estimated to die prematurely from smoking related illnesses. Both active and passive (second hand) smoking contributes to these high mortality rates with millions of smokers affected by a number of severe, smoking-related morbidities. In the US alone, these account for over $289 billion extra economic burden including ≈$133 billion for direct medical care and ≈$156 billion in productivity loss just for the years 2009–2012 [1].

Lung cancer (29 %) [3] and ischemic heart disease such as atherosclerotic diseases of the myocardium and blood vessels (28 %) [4] are the two major fatal morbidities directly associated with smoking. Chronic obstructive pulmonary disease (COPD) (21 %) and other forms of cancer (8 %) closely follow. Less common morbidities that have been recently linked to smoking include declined immune functions, rheumatoid arthritis, diabetes mellitus, eye diseases (such age related macular degeneration) and inflammatory bowel disease [5]. Focusing on cerebrovascular disorders, epidemiological studies have associated smoking with the pathogenesis and/or progression of a number of major neurological diseases. These include, but are not limited to, silent cerebral infarction (SCI) [6], stroke [7] and small vessel ischemic disease (SVID; due to the pro-coagulant and atherogenic effects of smoking) [8, 9] and cerebral aneurysms [10]. There is also a strong correlation between smoking and an increased risk for neurodegenerative disorders such as multiple sclerosis [11, 12], Alzheimer’s disease, and neurodevelopmental damage during pregnancy [13]. Although some of the neuropathological effects of TS seem to be dependent upon nicotine-activated specific pathways [14], the precise cerebrovascular harmful mechanisms triggered by TS remain largely unclear. However, recent studies clearly suggest that TS can trigger a loss of blood–brain barrier (BBB) function and integrity which is certainly a critical prodromal factor for the pathogenesis of these neurological diseases.

## Conceptual focus

In this review article, we will cover the current knowledge and experimental data concerning the direct effects of tobacco smoking at the brain microvasculature with a special focus on the TS impact on BBB physiology and function. We will also illustrate the determining toxicological factors of TS products including conventional and reduced exposure products such as electronic cigarettes. Using epidemiological evidence along with the limited body of direct toxicology studies, we then discuss the relationship of TS-induced toxicity with the increased risk and early onset of neurological/neurovascular complications in the smoking population. Next, we review current literature that highlights the role of anti-oxidant based defense mechanisms such Nrf2-ARE signaling as well as anti-inflammatory pathways in coping with TS-induced toxicity. In the same context, we will discuss the controversial role of antioxidant supplementation as a prophylactic and/or therapeutic approach to prevent the onset of disease complications or decrease their progression in smokers. Finally, we will provide insights on possible new avenues for future research including mechanistic studies involving direct inhalation rodent models.

## The blood–brain barrier

The BBB is mainly composed of microvascular endothelial cells (ECs) lining the luminal walls of the brain microvessels along with juxtaposed astrocytic end-feet processes and pericytes that support ECs differentiation and maintenance of BBB properties [15, 16]. BBB endothelium is functionally distinct from ECs in other vascular beds because they are characterized by little pinocytotic activity, absence of fenestrations (i.e., openings), and distinct distribution patterns of transmembrane transporters. These transporters strictly regulate the passage of nutrients and other essential elements while providing protection (e.g. efflux transporters) from possible harmful substances (both endogenous and xenobiotics). The strict tightness of the BBB ECs largely depend upon the presence of inter-endothelial tight junctions (such as zonulae occludentes-ZO-1, occludin, claudins, and junctional adhesion molecules-JAM) that form a physical barrier between adjacent endothelial cells thus, preventing the passage of hydrophilic substances through paracellular routes [17–19]. Another venue of entry across the BBB is controlled by asymmetrically distributed, carrier-mediated transport systems [20–22]. These allow the passage of water-soluble but biologically important substances (e.g., d-glucose, amino acids, monocarboxylic acids, etc. [16, 17, 22]) from the peripheral circulation into brain parenchyma. The space between the endothelial cells also features junctional complexes of adherens junction (AJ) proteins such as VE-cadherin. Clearly, loss of AJ leads to increased permeability [23] but these proteins primarily assist the TJ which are primary determinants of BBB tightness. The dominant functions of these proteins include cellular adhesion, contact inhibition and polarization of endothelial cells [24].

Apart from TJ and AJ proteins, specific efflux systems at the BBB (e.g., P-glycoprotein—P-gp [20], multidrug resistance associate protein 4—MRP4 [22] and breast cancer resistance protein-bcrp [25, 26]) limit the passage of potentially harmful amphipathic and hydrophobic substances by preventing their entry into the brain [20, 27]. These transporters work in concert with several drug metabolizing enzymes (including monoamine oxidases and cytochrome P450s) via activation of the pregnane X receptor (PXR) also known as the steroid and xenobiotic sensing nuclear receptor) [28, 29] to efflux the passage of harmful substances into the brain [17]. Although the expression and functional activity of these metabolic enzymes has not been quantified relative to other organs, their presence at the BBB endothelium has been postulated based on gene expression studies. For example, cytochrome P450 enzymes (e.g., P450 3A4) expressed at the BBB endothelial level under pathological conditions (e.g., drug-resistant epilepsy) [28, 29] were shown to actively metabolize carbamazepine into inactive derivatives in BBB endothelial cells isolated from brain tissue resections of drug resistant epileptic patients. In addition to xenobiotics, these P450 enzymes metabolize endogenous lipids and steroidal hormones [30].

## Impact of cigarette smoke on BBB and CNS

Cigarette smoking is considered a major risk factor for several neurological disorders and neurovascular complications including stroke, SVID and vascular dementia. Preclinical and clinical findings published so far attribute oxidative and inflammatory damage caused by a large and still poorly identified number of highly reactive oxidative species (ROS) contained in TS as the primary determinants of cigarette smoke-induced vascular toxicology. In addition, neurological complications such as Alzheimer’s disease, multiple sclerosis, stroke, small vessel ischemic disease and vascular dementia [31–38] also report the involvement of ROS and inflammation as central mechanisms initiating and promoting disease progression. Thus, it is a viable possibility that chronic smoking exacerbates the overall damage due to oxidative and inflammatory stimuli and predisposes the end consumer to these neuropathologies.

The BBB is rapidly exposed to this host of harmful toxicants and ROS present in TS which become a critical factor in TS-promoted CNS disorders. The BBB plays the crucial role of a dynamic interface which normally controls the passage of substances (both endogenous and xenobiotics) between the blood and the brain thus maintaining the brain homeostasis. When a cigarette puff is inhaled, a large number of soluble and gaseous components within the smoke rapidly pass through the lung alveoli into the arterial circulation (skipping first pass metabolism) and quickly reach the brain microvasculature. The brain parenchyma is effectively shielded from TS toxicants circulating in the blood by the BBB. However, chronic exposure to these substances may impact BBB viability and function overtime (e.g., lifelong chronic smokers). A functionally compromised BBB can then enable the onset and/or progression of neuroinflammatory and neurovascular disorders [39, 40] which in turn can kick off a vicious cycle of continued BBB impairment.

Despite the strong evidence of an association between smoking and vascular impairment, the impact of cigarette smoking on the BBB has only been marginally addressed. This is quite evident from the relative small number of basic and translational studies currently available in the literature. For example, the incidence of small vessel ischemic disease (SVID; a pathological condition characterized by loss of BBB integrity and leaky brain microvessels) in chronic smokers was shown to be significantly higher than non-smokers [41]. As a consequence of this disorder, patients typically manifest concerns such as gait problems, urinary continence and cognitive decline. The evidence of a leaky barrier in these patients was determined by measurements of S100β (a serum marker of blood–brain barrier integrity [41–44]) plasma extravasation and confirmed by Magnetic Resonance Imaging (MRI) scans showing widespread white and grey matter signals consistent with impaired BBB integrity [41].

*In vitro* toxicological testing of cigarette smoke using total TS particulate matter or soluble cigarette smoke extracts (CSE) is primarily focused on the lung and the cardiovascular system. However, the gaseous and soluble fractions quickly cross the lung alveoli, move into the arterial circulation and rapidly reach the cerebrovascular network (and the BBB) right away. Current BBB toxicological studies are limited to assessing the harmful impact of whole soluble TS extracts or nicotine; the main tobacco neurostimulant component.

**Fig. 1:**
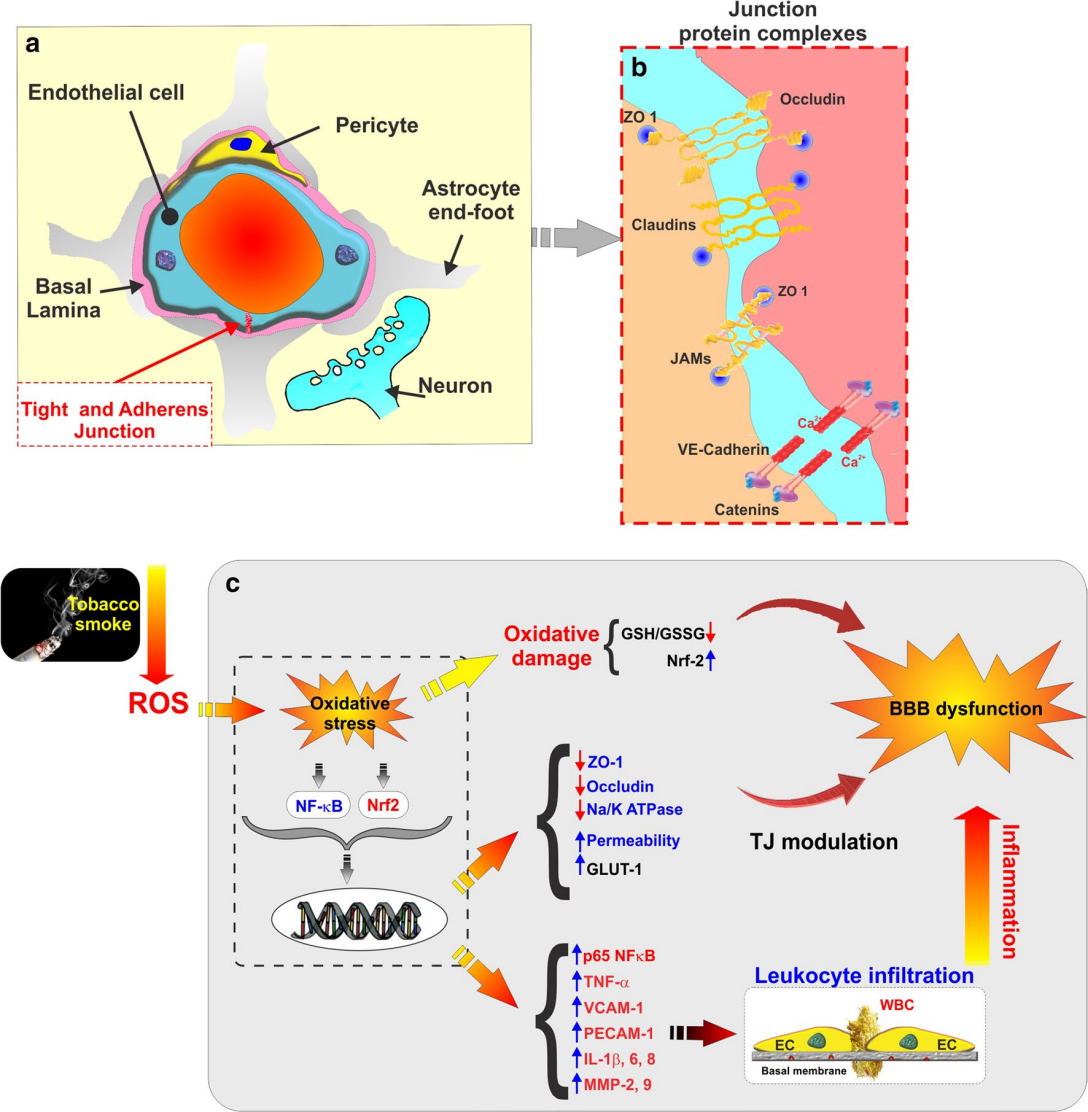
Schematic representation of the brain microvasculature features and corresponding Impact of TS: **a** Schematic illustration of a cross sectional view of a brain capillary. Note the endothelial cells are surrounded by supporting astrocytic end feet processes and pericytes along with the ensheathing basal lamina. **b** Paracellular passage of substances across the BBB endothelial layer is restricted by junctional protein complexes consisting of TJ proteins (such as occludin, claudins, JAMs); along with adherens junction protein VE-cadherin and catenins. Note that the cytoplasmic accessory protein ZO-1 intercalates these intercellular proteins with the cytoskeleton. **c** TS-produced ROS promotes oxidative stress responses at the BBB endothelium. These include the activation of several transcription factors including Nf-κB and the antioxidant response system via nrf2-ARE pathway. This latter in turn activates anti-oxidant and detoxification genes. The downstream effect of TS exposure leads to the down regulation of TJ proteins; increase in vascular permeability and activation of pro-inflammatory responses leading to BBB dysfunction

Previous work by our group using whole soluble TS extracts from research tobacco products (such as 3R4F; equivalent to conventional full flavor cigarettes) revealed a host of strong pro-inflammatory responses triggered by cigarette smoke at the BBB endothelial level [45]. The effect was significant both at the transcription and translational levels and included the up regulation of phase 1 and 2 detoxification mechanisms, activation of the antioxidant response pathways [46], up regulation of pro-inflammatory cytokines, vascular adhesion molecules and increased leukocyte-endothelial interactions [41]. This strong inflammatory response is crucially relevant to define the impact of TS at the cerebrovascular level since vascular adhesion molecules facilitate the adhesion of monocyte to ECs and extravasation across the BBB [47]. Moreover, pro-inflammatory cytokines play a major role in the pathogenesis and modulation of inflammation [48] and have been shown to regulate the trafficking of immune cells across the BBB into the brain by acting as modulator of cytoskeleton TJ proteins and actin filaments [49]. In fact, a direct assessment of the BBB endothelium revealed a significant down regulation of major TJ proteins such as ZO-1 and occludin paralleled by release of vascular endothelial growth factor-VEGF (a vasogenic factor that has been reported to play a major role in loss of BBB integrity [50]) and concomitant increase of paracellular permeability [46] (see also Fig. 1).

Apart from the whole soluble TS toxicants which seem to correlate well with oxidative stress generated by TS, nicotine exposure has shown to down regulate BBB endothelial tight junction protein expression such as ZO-1, occludin, cadherin, and adherens junctional proteins [51–53]. In a separate study by Abbruscato et al. nicotine exacerbated ischemic reperfusion (IR) injury and edema formation in experimental models of stroke [54]. Interestingly, the investigators observed a decrease in Na^+^/K^+^/2Cl^−^ co-transporter activity following IR with prior nicotine and/or tobacco smoke exposure [52, 55]. Furthermore; nicotine has been shown to promote angiogenesis in vitro in HUVECs and HCAECs mimicking the effects of VEGF thus increasing capillary density and stimulating the growth of collateral blood vessels in mouse models of hind limb ischemia [56].

A noteworthy finding is the impact nicotine has on the drug disposition of saquinavir- an anti-retroviral drug used in Human Immunodeficiency Virus (HIV) therapy. The study reported that chronic exposure to nicotine (delivered via subcutaneously osmotic pumps) impaired the activity of efflux transporters such as P-gp along with the loss of TJ protein ZO-1 and Notch-4 expression [57, 58]. However, chronic nicotine exposure did not impede the passage of polar paracellular markers in sucrose perfusion studies. The same group also reported alterations in passive permeability or diffusion of compounds with low extraction values [59]. Although detailed in vivo studies, based on chronic exposure of total whole soluble TS toxicants and their impact on the BBB function and efflux transporters are warranted; so far the reported studies clearly indicate the overall potential of TS toxicants to impact BBB function.

Regarding the pro-inflammatory activity of TS, elevated levels of leukocytes are commonly observed in smokers [60]. In particular, neutrophils, which secrete free radicals, elastase and collagenase [61], are thought to contribute directly to EC injury. Platelet activation is also frequently observed [62], as confirmed by in vitro and in vivo studies [63]. In conformity to a previous report by Nordskog, release and activity of matrix metalloproteinase-2 and -9 [64] was also significantly increased. MMP-2 and 9 primarily target the BBB by degrading components of the basal lamina and facilitating immune trafficking into the brain [65]. TS directly promoted the differentiation of monocytes into macrophages independently from the presence of activated endothelial cells. Metalloproteinases such as MMP-9 are known to directly affect BBB integrity and have also been reported in immune cell trafficking [66, 67]. The importance of these inflammatory events has been described in both in vivo and in vitro settings with the use of human aortic endothelial cells [68, 69]. Together with local infiltration and activation of circulating immune cells, these processes may contribute to the pathogenesis of vascular inflammatory disorders which have been linked to the onset and/or progression of several neuroinflammatory and neurovascular diseases [70, 71]. Previous studies from our group have also shown that tobacco smoke exposure impacts BBB endothelial inflammatory response at the gene transcriptional level [41, 46]. In fact, transcription of inflammatory modulators such as NF-kappaB, RelB and STAT3 (which is also an angiogenesis modulator [72, 73] and a molecular linker for extracellular signals to transcriptional control of proliferation and immune evasion) were significantly upregulated by TS. Furthermore, SAA1 (a potent chemoattractant factor also responsible for the transcription of amyloid A [74]) and APOE (directly related to atherosclerotic diseases and ischemic damage [75]) gene expression were also upregulated. Specifically APOE is responsible for the production of apolipoprotein E, which is essential for the normal catabolism of triglyceride-rich lipoprotein constituents. This polymorphic gene has been studied for its role in several biological processes related to immunoregulation and is associated with elevated cholesterol and risk of atherosclerosis and ischemic stroke.

Further, TS contains high concentrations of nitric oxide (NO) [76], which also can affect BBB integrity. NO plays critical role in controlling vascular tone, leukocyte-endothelial adhesion and platelet aggregation. NO has been shown to modulate BBB function [77, 78] and is directly involved in a number of pathological processes inherent to inflammation. NO has a vasodilatory effect during the early stage of ischemic injury which appears to be protective for the brain [79]. This is early stage endothelial response promoted by eNOS activity is soon followed by activation of inducible NO synthase (iNOS) in inflammatory cells infiltrating the brain as well as the brain microvasculature leading to a massive spike of NO production (which peaks at 12–48 h after ischemia). Through a process of redox cycling NO is diverted toward the formation of peroxynitrite followed by production of superoxide anion radicals O_2_^−^ [80] which propagates inflammation to neighboring districts propagating the damage. This ultimately results in the initiation and progression of vasculo-pathogenic diseases such as atherosclerosis, thrombosis.

Currently, vascular pathogenic processes activated in response to chronic tobacco smoking are far from being fully understood. Although, inflammation as well as oxidative damage have been shown as major determinants of TS toxicity in studies published so far, additional studies both in vitro and in vivo will be necessary to break down the molecular targets and mechanisms involved. This is of critical importance in lieu of recent mechanistic studies outlining the parallel between cerebrovascular toxicity of tobacco smoke, its oxidative potential and the concurrent stimulation of a major antioxidant response system; the Nrf2-ARE pathway [46].

## Nrf2-ARE Pathway in cigarettes smoke-induced oxidative stress

Nuclear factor (erythroid derived 2) like 2 known as NFEL2 or Nrf2 is a cap’n’collar (CNC) basic-region leucine zipper (bZIP) transcription factor which plays a major role in countering oxidative stress [81–83]. Under basal conditions it is usually maintained at a low level in cytoplasm and bound to keap-1 which directs it to proteasomal degradation. Gene polymorphisms in the promoter region, post transcriptional modifications and protein–protein interactions have also been reported to modulate Nrf2 basal activity [84]. Of interest in the current context, however, is the inducible response of Nrf2 by TS-induced oxidative stress via ROS generation.

Under an oxidative stress insult, keap1 is ubiquitinated and Nrf2 becomes free to translocate from its cytoplasmic subcellular location into the nucleus. Upon binding to the antioxidant response element (ARE), it activates the transcription of several downstream genes mainly involved in detoxification and antioxidant processes [83, 85]. The major downstream effectors of the ARE pathway can be classified into the following main categories: (1) detoxification system including Phase I (oxidation/reduction), Phase II (conjugation enzymes) and Phase III (drug efflux transporters) [83, 85]. The Phase I genes are involved in oxidation/reduction/hydrolysis biochemical functions including enzymes belonging to the aldo–keto reductase family, aldehyde and alcohol dehydrogenases, cytochrome P450, NAD(P)H: Quinone reductase I (NQO1) and carbonyl reductase (CR) to name a few. Substances then enter Phase II where they are conjugated to bulky polar groups such glutathione, glucoronic acid, sulfate or glycine and converted into more readily excretable forms. Apart from these biochemical reactions, potentially harmful compounds are also kept out of the cell through the activity of efflux transporters belonging to the ATP binding cassette (ABC) family; (2) antioxidant system (including glutathione—GSH and thioredoxin); (3) heme and iron metabolism; (4) Carbohydrate and lipid metabolism; (5) cross talk between transcription factors.

Many studies report high transcriptional activity of these factors in several disease models of acute and chronic oxidative or inflammatory injuries in the brain as well as other peripheral organs. These include disorders related to oxidative and/or inflammation such as diabetes [86, 87], ischemia reperfusion injuries [88, 89], cardiomyopathies and heart failure [90], liver fibrosis [91], and chronic kidney disease [92]. Activation of the Nrf2 pathway and up regulation of several downstream effector proteins has also been reported specifically due to cigarette smoking on resident macrophages, lung bronchial and alveolar epithelium and lung fibroblasts of chronic smokers [93]. Nrf2 activation has been shown to play a major coping role against the onset and progression of COPD and emphysema- major disorders associated with smoking [81, 94, 95].

Unfortunately, the vast majority of current literature concerning the mechanistic details of TS toxicity primarily covers the pulmonary and cardiovascular systems leaving the cerebrovascular system poorly understudied. This remains a critical issue to be addressed since the importance of the Nrf2-ARE pathways at the brain microvascular level have been clearly emphasized by a number of recent studies focused on cerebrovascular oxidative stress injuries. These injuries include stroke (both global and focal ischemia) [96], subarachnoid brain hemorrhage [97], amyotrophic lateral sclerosis (ALS) [98], multiple sclerosis (MS) [99] and Alzheimer’s disease (AD) [100] and diabetes [101, 102]. Interestingly, the impact of Nrf2 signaling has been reiterated in several recent, preclinical studies which have clearly shown that boosting antioxidant pathways through Nrf2 enhancers or antioxidant supplements such as docosahexaenoic acid (DHA), resveratrol, bicyclol can be beneficial in neuropathologies such as cerebral stroke [103–105]. Nrf2 has shown to play a cytoprotective role against TS exposure in BBB endothelial cells. Our group has observed nuclear translocation of Nrf2 followed by increased transcription (and translation) of detoxification enzymes and anti-oxidants in response to TS exposure [46].

In contrast, a number of clinical and preclinical studies concerning the effects of chronic smoke have revealed that the organs exposed to TS manifested a defective and compromised Nrf2 signaling [106, 107]. One such study highlighted the inhibition of the Nrf2/ARE pathway due to cigarette smoking in peripheral mononuclear cells of young heavy smokers which promoted inflammation and exacerbated damage [108].

Unfortunately, to date, the pathobiology of cigarette smoking at the brain and brain microvascular level is still poorly understood. How these pathways are activated and if chronic TS exposure can impact Nrf2-based mechanisms operating at the BBB level is a question still open for investigation. It is therefore evident that there is urgent need to identify new avenues of intervention for reducing the risk of cerebrovascular disorders in smokers and, perhaps, accelerate the recovery of the antioxidant system during smoking cessation.

## Antioxidant supplementation in smokers: where do we stand today?

Tobacco smoke generates superoxide and other reactive oxygen species which promote DNA strand breakage [109–112], release of nitric oxide (NO) and antioxidant depletion (e.g., ascorbic acid). Under normal conditions, ROS are cleared by the intracellular action of superoxide dismutase (SOD), catalase, glutathione (GSH) peroxidase [113] or extracellular antioxidant vitamins such as ascorbic acid (vitamin C), and α-tocopherol (vitamin E) [114–117]. However, environmental factors including active and passive TS spawn sustained high levels of ROS beyond the ability of the human body to effectively eliminate them. In fact, several studies have shown that chronic smokers suffer from antioxidant shortages caused by increased anti-oxidative mobilization evoked by TS [118–120]. Over time (e.g., chronic smokers) this imbalance is likely to promote oxidative damage both to cells and tissues. A recently published study by our group [45] has demonstrated that TS contains high concentrations of NO and ROS leading to the initiation and progression of various vasculopathies (e.g., atherosclerosis, thrombosis) as well as BBB damage. Indeed, the current scientific opinion considers ROS-mediated pathways to contribute significantly to the pathogenesis of many neurological diseases. This hypothesis is strongly supported by in vivo and in vitro experiments where antioxidant supplementation prevents oxidative damage and inflammation induced by cigarette smoke. Even the Food and Nutrition Board of the National Academy of Sciences has established a higher recommended dietary allowance (RDA) of vitamin C for smokers (over 200 mg/day versus the recommended 90 mg/day for non-smokers). However, clinical studies have shown a number of contrasting results with in vitro and/or in vivo studies regarding the therapeutic effect of antioxidants in a number of neurovascular/neurodegenerative disorders [121–125]. This makes it challenging to argue for or against the prophylactic and/or therapeutic use of anti-oxidants in smokers. Recent observations suggest that ROS are key mediators of BBB breakdown [126] and antioxidant supplementation has proven to be beneficial in alleviating a loss of BBB integrity and a vascular inflammatory response in smoke-exposed in vitro BBB cultures [127]. Although there is still no unequivocal evidence that an increased intake of antioxidant nutrients can effectively counteract TS toxicity, there is supporting data suggesting that antioxidants may prove to be effective scavengers of exogenous (TS-like)-derived ROS [128]. For example, vitamin C prevents histamine release and increases the detoxification of histamine [129], thus acting as an anti-inflammatory agent as well as a potent antioxidant. Vitamin E on the other hand has been shown to be cardioprotective against tobacco smoke-induced peroxidative damage [130–132] and can be a beneficial adjuvant in the treatment of seizures, diabetes and in the reduction of post-ischemic damage [133–136]. Recently published in vitro studies by our lab have clearly shown that both vitamin C and E can effectively protect the BBB against TS-generated oxidative damage [127]. Nevertheless, at this point the use of antioxidants (including Nrf2 enhancers) needs to be considered with caution. To illustrate this, in most cases there is not a far reaching consensus in the clinical setting regarding dosing parameters, (including frequency of administration). Recently published preclinical studies also indicate that cancerous cells prioritize Nrf2 activation to promote their survival from antioxidant damage [137, 138]. Furthermore, high-mobility group protein B1 (HMGB1; a mediator of inflammation produced by necrotic tissue and activated immune cells) appears to be involved in the post-ischemic inflammatory response and has been correlated to poor functional outcome [139–141]. Because the redox state of the intra- and extracellular environments control the activity of HMGB1-mediated pro-inflammatory signaling [142, 143], post-ischemic administration of antioxidants (therapeutic administration) may instead prolong and intensify the pro-inflammatory stimulation at the site of injury by neutralizing the ROS required to abate HMGB1 activity. Considering these premises, it is clear that more detailed and well-designed/standardized studies will be necessary to solve this impasse.

## Working around cigarette toxicity from an industry perspective

### Reduced exposure cigarette products

Cigarette smoke consists of about 7000 different chemicals and potential toxicants which may be included in either the gas and/or the particulate fractions of TS. Tar is defined as the dry solid residue deriving from the combustion of tobacco which yields the particulate fraction of cigarette smoke. This is often termed as TPM which contains nicotine as well as numerous carcinogens, chlorinated dioxins, furans metals, poly aromatic hydrocarbons (PAH), nitrosamines, terpenoids, and paraffin waxes [144, 145]. The gas phase of cigarette smoke includes gases of combustion such as carbon dioxide (CO_2_), carbon monoxide (CO), nitrogen (N_2_), oxygen (O_2_), hydrogen cyanide (HCN), hydrogen sulfide (H_2_S), nitric acid, acetone, acrolein, acetaldehyde, methane, ammonia, methanol, along with hydrocarbons, gas phase nitrosamines [(N-nitrosoanabasine (NAB), N-nitrosoanabatine (NAT), 4-(methylnitrosamino)-1-(3-pyridyl)-1-butanone (NNK), and nitrosonornicotine (NNN)] and carbonyl compounds [144]. In addition, exposure to these substances can increase intracellular levels of ROS through enhanced mitochondrial activity [146–149]. This ultimately can lead to the formation of adducts at lipids, proteins and DNA level such as 4-hydroxy-nonenal (4-HNE) and lipid peroxidation products [150–152], protein carbonyls [153, 154] and DNA adducts [150, 155] respectively.

Due to the complex nature of identifying and determining these toxicants, which may vary according to the fabrication procedure of the various cigarette brands and lack of central control in their manufacture, the FDA initiated an independent center dedicated to the production and distribution of standardized research tobacco products (reference cigarettes) in 2009 reflecting the main cigarette denominations currently available in the market (e.g., 3R4F—full flavor; 1R5F, light flavor). The tobacco industry has developed “reduced exposure” and “light” cigarettes containing lower levels of nicotine, nitrosamines or other chemicals deemed to be potentially toxic. However, the underlying claim that these products are safer than conventional cigarettes is not supported by experimental and/or clinical data. Recent smokeless tobacco products are marketed to current and “potential” smokers as a safer alternative to conventional products. At the same time, these “light” products also contain a certain amount of tobacco specific nitrosamines. There is a significant difference between these “light” cigarettes versus products that deliver tobacco-free nicotine, such as nicotine replacement therapies (NRT’s, e.g., nicotine gum, lozenge and inhaler) or electronic cigarettes (e-cigarettes).

Recent reports using whole soluble TS extracts from conventional and reduced exposure products (including ultralow nicotine—ULN—cigarettes obtained from National Institute of Drug Abuse-NIDA) revealed that the total oxidative and nitrosative capacity as well as pro-inflammatory activity of ULN (a cigarette containing negligible nicotine but quantities of tar comparable to conventional full flavor products) and nicotine free- NF (non-tobacco based) cigarettes were comparable or even worse than conventional cigarettes [45] thus negatively affecting the brain microvascular endothelium [45, 46, 156]. This creates quite a confusion as to what extent and in which form these reduced exposure products are to be considered safer than their conventional counterparts.

## E-cigarettes

**Fig. 2:**
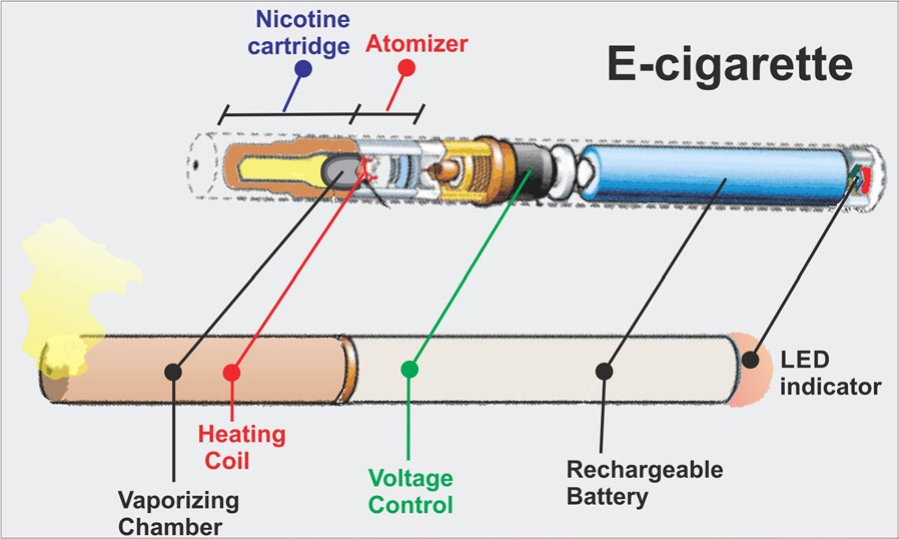
Schematic illustration of a typical e-cigarette: This device mainly consists of a cartridge containing nicotine dissolved in an e-liquid which also contains flavoring agents; an atomizer that vaporizes the e-liquid and a rechargeable battery (sometimes with a LED indicator) that provides power to the atomizer. The atomizer and the cartridge are sometimes collectively called the cartomizer. The battery output and the resistance of the heating coils determine the vaping capacity of the device

Electronic (e-) -cigarettes are members of the recently marketed electronic nicotine delivery devices (ENDDs). E-cigarettes can be primarily described as electronic, smokeless, nicotine delivery systems simulating cigarette smoking independently of the combustion of tobacco. There are at least 400 different brands of e-cigarettes currently available in the market (see Table 1 for a list of brands) [157]. E-cigarettes contain a fluid-filled cartridge (including numerous ingredients such as vegetable glycerin (VG) responsible for the visible vapor, propylene glycol (PG) serving as a flavor diluent, nicotine, menthol and other flavoring agents), an atomizer (which vaporize the e-liquid by heat) and a power source consisting of a rechargeable battery that charges the atomizer (see Fig. 2). Puffing on the e-cigarette vaporizes the fluid, allowing for the appearance of a “vape” which is delivered to the airways and from there across the lung alveoli into the circulatory system. To date, about three generations of e-cigarette designs have come up in the market with higher battery capacities, more heating power and sophisticated models although relying on the same basic components and principles highlighted above.

**Table 1:** List of brand of e-cigarettes currently available in market

E-cigarettes	Design features	Popular e-cigarette products under the Brand
Vapor Fi	Two-piece sophisticated designs Selection of refillable tank-style e-cigarettes with good vapor production Offers over 30000 flavors Range of tank sizes and battery power Variable voltage/airflow	Express starter and Pro starter kit for beginners VaporFi Rocket—for more experienced users 1 Rocket Tank with 1 dual coil and fully adjustable airflow control Vox II mod—stronger vape 50 watts of power vaporizer (adjustable)
V2 Cigs	Two-piece design with disposable as well as refillable versions Newer line of product designs available includes the V2 Pro Makes their own e-liquid with 24 flavor options Provides battery options with both manual as well as automatic version	EX line of e-cigarettes are the top miniatures, pen-style e-cigarettes V2 Pro series Cartridge recognition to optimize the temperature of the atomizer Can vaporize three types of ingredients V2 Disposables and Zig-Zag^Tm^ are disposable e-cigarettes
Green smoke	Two-piece design disposable cartomizer system in a range of flavored cartridges Designed for beginners	FlavorMax^Tm^ cartridge holds the e-liquid and the unit Available in different sized starter kits and packs
Halo cigs	Halo Cigs offer a well-built product with mainly two designs (Halo G6 e-cig. and Triton Vape pen) Makes their own e-liquid	Halo G6 rechargeable e-cigarette Leak proof and refillable e-cig cartomizers Different size tank options Triton vape pen e-cig with a leak-proof vape tank Variable voltage long-lasting batteries
Apollo	Advanced clearomizer (cartomizers with clear bodies) technology Makes their own e liquid (25 flavors available); Refillable as well as disposable versions Range of battery options from low to higher power output	Extreme kit (low battery power for new users) Endevour kit (Intermediate battery power) Vtube kit (maximum vape/battery power with variable voltage option; generally, for heavy smokers) Apollo Disposable and E-cigar Disposable products
Blu Cigs	Two-piece sleek design in disposable or prefilled designs (with blu Tank^Tm^ or flavor cartridges) Signature blue LED tip: Lights up to let you know that your blu e-cig is working Silicone tip: Intake maximizes each draw	Blu rechargeable e-cigarette (blu flavor cartridge with rechargeable battery that charges in the USB chargeable pack); PLUS + rechargeable™ (6-hole tip, more powerful PLUS + rechargeable battery and blu™ tank) Blu™ disposable electronic cigarettes
EverSmoke	Looks and feel like a real cigarette Two battery sizes and range of flavored cartridges with a Silicone Tip VaporFlo™ technology for smooth draw	EverSmoke Electronic Cigarette (tobacco, menthol and other flavored rechargeable cartridges) Available in different sized starter kits and packs

Nicotine, the main vape component, is present in the maximum range of 20 mg/ml [158]. The vapor formation after heating of the e-liquid is not consistent. It is dependent on the product design, and puffing parameters including puffing rate/duration/volume. Nevertheless, the absorption of nicotine is reported as considerably less than conventional “combustion-based” counterpart products. Other major components of the e-liquid are propylene and diethylene glycol. Release of these glycols in the vapor is estimated to be with-in safe limits based on some recent reports [159]. However, whether they undergo further physicochemical modification (by the heat necessary to vaporize the e-liquid) into more harmful compounds has not been investigated. Further, there are no defined parameters and established guidelines concerning preparation of the e-liquids and their composition which adds serious safety concerns regarding e-cigarettes.

Increasing the market appeal is their wide range of flavoring agents that can be added to the e-liquid mix such as vanillin, cinnamon, various fruits, menthol, etc. to name a few. These flavoring agents which are proven safe for use in food or confectionary products (ingested) are assumed to be equally safe when vaporized in the form of e-liquids (inhaled). Limited but steady evidence clearly suggests that these flavoring chemicals are present beyond the National Institute for Occupational Safety and Health (NIOSH) safety range and can be a potential irritant when directly inhaled [159–162].

## Toxicological data currently available about e-cigarettes

First introduced into the consumer market in 2005, the global market for ENDDs has rapidly expanded. It has been predicted that within the next decade, sales of ENDDs will surpass that of traditional tobacco-based cigarettes. In 2011, it was estimated that 21 % of US adult cigarette smokers had tried an ENDD [163] which are also quite popular among adolescents and young adults [164]. The rapid rise of ENDD use has divided the public health and tobacco control community [165, 166]. Although there is potential for public health harm reduction through decreased use of combustible tobacco products [167, 168], there are significant concerns about the limited scientific information with respect to the short and long-term effects on human health as well as their intrinsic potential to attract new and former smokers. A high percentage of former smokers are reported to choose e-cigarettes in an effort to quit smoking and/or to allow for smoking in situations where traditional cigarettes are not allowed [169] due to the popular perception of their relative safety [170–173]. Thus, the addictive potential of e-cigarettes is also a major concern.

A critical barrier for the Food and Drug Administration’s (FDA’s) Center for Tobacco Products (CTP) regulation of all types of cigarettes is the identification of constituents that are harmful to human health. Sadly enough, clinical and preclinical studies supporting the conceptual claims that e-cigarettes are significantly safer than conventional tobacco products are not backed by a body of research evidence. There are a handful of toxicological studies on e-cigarettes that have mainly focused on limited cytotoxicity assessments without considering the detailed vapor composition inhaled by the end-user [161]. Moreover, these studies are limited and to date, have only been performed in vitro on a limited number of cell phenotypes such as pulmonary fibroblasts, bronchial epithelial cells, embryonic stem cells, and neural stem cells [174–176]. Although these reports suggest e-liquids to be safe, there are some observed differences in cytotoxicities attributed to the flavors used. Apart from cytotoxicity assays there are also some chemical assay reports suggesting that, in respect to the content of tobacco specific nitrosamines (TSNAs), carbonyls, volatile organic compounds (VOC) and heavy metals, e-cigarettes are relatively safer [177]. However, analyses of the e-liquid revealed the presence of residual aromatic hydrocarbons, formaldehyde, acetone, minor tobacco metabolites (e.g., anabasine, myosmine, β-nicotyrine), propylene glycol, diethylene glycol, and tobacco specific nitrosamines including carcinogens in a wide range of concentrations, thus suggesting a lack of standardization of the raw materials and/or manufacturing processes [159, 161]. Further, some constituents of e-cigarette vapor (e.g., flavoring agents) not present in conventional tobacco smoke have been shown to be cytotoxic in embryonic/adult cellular models [178] and/or represent a possible environmental hazard such as copper [179].

Thus, there are many brands of e-cigarettes currently available in market (as illustrated in Table 1). Due to lack of current regulatory systems each brand produces its own e-liquids which differ for composition, including flavoring agents, nicotine concentration, solvent constituents such as propylene glycol and vegetable oils as well as different levels of heating power. These many variabilities in e-cigarette brands may directly impact the toxicity profile of the final products. These warrant for comparative toxicological studies which will be necessary to inform the FDA and generate common manufacturing guidelines. In this regard, the most recent safety studies have been limited to the analysis of heating byproduct derivatives and corresponding direct toxicological assessments (both qualitative and organ specific) [179–181]. Direct vaping of the e-liquid and inhalation studies using preclinical animal models first requires standardization of methods and delivery of the vape to mimic common inhalation patterns of e-cigarettes in the end user. Only then can short and long term toxicological studies on e-cigarettes be reliably planned and executed. Moreover, these toxicology studies need to address not only the primary sites of inhalation (such as the oral cavity, lung mucosa and the cardiovasculature) but also should be extended to brain and the brain microvasculature. Such preclinical toxicological studies are essential to support the regulatory authorities and to set quality standards of this product with the concern of public health in mind.

## Unknown health impacts and new regulatory challenges regarding e-cigarette

The Family Smoking Prevention and Tobacco Control Act (FSPTCA) gave the FDA the authority to regulate the manufacture, marketing and distribution of tobacco products. This authority includes the review of new and modified tobacco products prior to their introduction to the market and to establish standards for tobacco products. A recently conducted worldwide survey found that both former smokers and current heavy smokers initiated e-cigarette use based on the perceived benefits of lack of toxicity and negligible effects of second hand smoking exposure for their families [182] versus the use of regular products. These surveys clearly indicate the end-consumer is making serious health decisions based on the commercial claims of these products. Consumers assume that e-cigarettes prolong abstinence and/or promote a more effective smoking cessation program than RTS. However, current clinical studies are inconclusive [183, 184] and, therefore, e-cigarettes provide uncertain benefit in quitting smoking. The CTP division of the FDA currently monitors and reviews cigarettes, roll-your-own tobacco and smokeless tobacco. However, due to the alarming concern over the use of e-cigarettes, it initiated public forums to report adverse events and toxicities associated with the use of new electronic devices. In addition, it has recently proposed a rule to extend the regulation of these newly “deemed” products (such as e-cigarettes) which are incorporated under CTP regulatory monitoring.

## Conclusion and future perspectives

Although, epidemiological evidence and clinical studies have clearly shown that tobacco smoking is a major risk factor for the pathogenesis of several neuro-inflammatory and neurovascular disorders [6, 185, 186], detailed toxicological and mechanistic studies focused on TS effects at the brain and brain microvasculature are quite scarce. The few basic studies addressing this crucial issue have shown that TS exposure is likely to impact BBB physiology and functions by promoting oxidative stress damage and inflammation. Although indicative of the potential TS toxicity at the BBB, these studies have been limited at large to in vitro settings following acute or limited chronic exposure which may not fully recapitulate the complex dynamics of a physiological setting. Studies in vivo have been limited to a handful of constituents (mainly nicotine) contained within the several thousands of compounds found in TS. Therefore, there is a clear lack of knowledge in regard to TS cerebrovascular toxicity that needs to be addressed. Direct chronic smoke inhalation studies in vivo along with assay of the additional physiological alterations (e.g., blood hemostasis, neuroinflammatory biomarkers, etc.) will be necessary to gather realistic data in a setting that more closely mimics the smoking patterns of the end user. Additional mechanistic insights will enable us to elucidate the antioxidant as well as inflammation based cytoprotective mechanisms at the BBB level and their overall capacity to sustained the oxidant load generated by TS as well as other oxidative stimuli. This will take us a practical and important step forward in understanding the health risk associated with tobacco smoking regarding the onset of neurovascular and neuro-inflammatory diseases such as cerebrovascular stroke, diabetes, Alzheimer’s disease, SVID, vascular dementia and multiple sclerosis. These data could be also used to identify a number of putative prognostic biomarkers to assess the smoker risks for the pathogenesis and/or progression of neurological disorders. Apart from these studies largely focused on the mechanistic component, therapeutic studies aimed at the betterment of health outcomes of the smoking population are equally crucial. In that direction, the use of Nrf2 enhancers has demonstrated impressive results to improve cerebrovascular pathologies such as stroke outcomes [103, 187]. Nrf2 driven activation of ARE pathway may be compromised in a BBB that is chronically exposed to tobacco smoke (TS); which in an event of cerebrovascular ischemic insult may lead to exacerbated loss of BBB integrity/function and secondary brain injury. These enhancers (or activators) can potentially benefit the health of the smokers through improved anti-oxidant capacity along with smoke cessation or reduction aids currently available in the market.

Concerning reduced exposures tobacco products, currently available toxicological studies examining the cerebrovascular system and the CNS are very scarce. For products recently introduces in the consumer marker such as e-cigarettes, the lack of toxicological data is even more dramatic considering that the very limited number of studies published so far focus most exclusively on the respiratory system. In addition, standardized toxicological testing paradigms to compare e-cigarettes versus traditional tobacco products have not been developed. The urgency of filling this gap is strongly dictated by a number of population-based studies suggesting that the use of e-cigarettes (especially among young individuals) will soon surpass that of conventional cigarettes. Enforcement of Good Manufacturing Practices is also a “must” to ensure quality standards in the preparation of the tobacco products including e-liquids and safety of the main raw materials utilized.

## Abbreviations

CO: carbon monoxide
N_2_: nitrogen
4-HNE: 4-hydroxy-nonenal
ABC: ATP binding cassette
AD: Alzheimer’s disease
ALS: amyotrophic lateral sclerosis
ARE: antioxidant response element
BBB: blood–brain barrier
Bcrp: breast cancer resistance protein
CO_2_: carbon dioxide
COPD: chronic obstructive pulmonary disease
CR: carbonyl reductase
CTP: center for tobacco products
DHA: docosahexaenoic acid
e-cigarettes: electronic cigarettes
ECs: endothelial cells
ENDDs: electronic nicotine delivery devices
FDA: Food and Drug Administration
FSPTCA: family Smoking Prevention and Tobacco Control Act
GSH: glutathione
H_2_S: hydrogen sulfide nitric oxide
HCN: hydrogen cyanide
HIV: human immunodeficiency virus
HMGB1: high mobility group protein B1
IR: ischemia reperfusion
JAM: junctional adhesion molecules-
MRI: magnetic resonance imaging
MRP4: multidrug resistance associate protein 4
MS: multiple sclerosis
NAB: N-nitrosoanabasine
NAT: N-nitrosoanabatine
NF: nicotine free
NFEL2 or Nrf2: nuclear factor (erythroid derived 2) like 2
NIOSH: National Institute for Occupational Safety and Health
NNK: 4-(methylnitrosamino)-1-(3-pyridyl)-1-butanone
NNN: nitrosonornicotine
NO: nitric oxide
NQO1: NAD(P)H: quinone reductase I
NRTs: nicotine replacement therapies
O_2_: oxygen
PAH: poly aromatic hydrocarbons
PG: propylene glycol
P-gp: P-glycoprotein
PXR: pregnane X receptor
RDA: recommended dietary allowance
ROS: reactive oxidative species
SCI: silent cerebral infarction
SVID: small vessel ischemic disease
TPM: total particulate matter
TS: tobacco smoke
TSNAs: tobacco specific nitrosamines
ULN: ultralow nicotine
VEGF: vascular endothelial growth factor
VG: vegetable glycerin
VOC: volatile organic compounds
ZO-1: zonulae occludentes
